# Fairness: the hidden challenge for competency-based postgraduate medical education programs

**DOI:** 10.1007/s40037-017-0359-8

**Published:** 2017-05-17

**Authors:** Colleen Y. Colbert, Judith C. French, Mary Elizabeth Herring, Elaine F. Dannefer

**Affiliations:** 1 Cleveland Clinic Lerner College of Medicine, Case Western Reserve University, Cleveland, OH USA; 2 Department of Medicine, Cleveland Clinic, Cleveland, OH USA; 3 Department of General Surgery, Cleveland Clinic, Cleveland, OH USA; 4 Department of Humanities, Texas A&H HSC College of Medicine, Bryan, TX USA

**Keywords:** Fairness, Assessment, Postgraduate medical education, Competency based medical education

## Abstract

Competency-based medical education systems allow institutions to individualize teaching practices to meet the needs of diverse learners. Yet, the focus on continuous improvement and individualization of curricula does not exempt programs from treating learners in a fair manner. When learners fail to meet key competencies and are placed on probation or dismissed from training programs, issues of fairness may form the basis of their legal claims. In a literature search, we found no in-depth examination of fairness. In this paper, we utilize a systems lens to examine fairness within postgraduate medical education contexts, focusing on educational opportunities, assessment practices, decision-making processes, fairness from a legal standpoint, and fairness in the context of the learning environment. While we provide examples of fairness issues within US training programs, concerns regarding fairness are relevant in any medical education system which utilizes a competency-based education framework.

Assessment oversight committees and annual programmatic evaluations, while recommended, will not guarantee fairness within postgraduate medical education programs, but they can provide a window into ‘hidden’ threats to fairness, as everything from training experiences to assessment practices may be examined by these committees. One of the first steps programs can take is to recognize that threats to fairness may exist in any educational program, including their own, and begin conversations about how to address these issues.

## Introduction

The primary goal of competency-based education systems is to provide educational and assessment experiences that allow learners to hone skills, increase knowledge, and enhance abilities via frequent formative feedback in order to meet targeted outcomes [1–7]. Theoretically, competency-based education systems, with their increased emphasis on criterion or standards-referenced assessment, allow institutions to individualize teaching practices to meet the needs of diverse learners [7–9]. Learners, in turn, are expected to take more responsibility for their own learning [5, 10, 11]. That said, all learners must ultimately meet outcomes set by the program, the institution and, in the case of postgraduate medical education programs, by accreditation agencies in their home countries. Yet, we argue that the focus on continuous improvement and individualization of curricula has not exempted programs from treating learners in a fair manner [12–14]. When learners fail to meet key competencies and are placed on probation or dismissed from training programs, they may seek legal redress; issues of fairness often form the basis of these legal claims [12, 14, 15]. While the notion of fairness has been mentioned, [16, 17] we found no in-depth examination of fairness in a search of the literature on postgraduate medical education (Appendix). In this paper, we utilize a systems lens to examine fairness within postgraduate medical education contexts, focusing on educational opportunities, assessment practices, decision-making processes, legal considerations, and fairness in the context of the learning environment. Although we provide examples of fairness issues primarily within US programs, concerns regarding fair treatment of trainees apply to any educational system which utilizes a competency-based education framework.

## Background

### Defining fairness

Cole and Zieky [18] noted that until the 1960s there were few professionals in the fields of educational and psychological testing and measurement who were concerned with issues of fairness. By the 1970s, there was a renewed interest in fairness, with research focusing on prevention of test bias [18]. Despite no universal agreement on definitions of fairness within educational contexts, [18–20] fairness within the testing literature is often viewed as ‘equal’ practice or treatment [19]. An outgrowth of fairness concerns in testing is the use of standardized tests, such as in-training exams and board exams [19–21]. Yet, this satisfies only one definition of fairness: equality or equal treatment of trainees [21, 22]. Equal treatment, unfortunately, does not always ensure fairness [21, 22]. In the field of educational testing, the definition of fairness now also includes the notion of equity [19–21, 23]. Equity in assessment practices first ensures trainees have comparable opportunities to *learn*, and then demonstrate new knowledge and skills, given their unique backgrounds [20, 22, 23]. Similar outcomes for subgroups, such as female trainees in male-dominated specialties, would be one indicator of fair treatment from an ‘equity’ perspective [18–20]. For the purposes of this paper, we have defined fairness as equity (e. g., comparable opportunities) and equality (i. e., equal or identical treatment). We provide examples of both throughout the paper.

### Access to comparable educational opportunities

A fundamental question postgraduate medical education training programs will need to ask at least annually (if not more frequently) is: did all trainees have access to comparable curricular experiences, allowing them to meet all of the program’s competencies [14, 19, 24]? While competency-based education frameworks require that all trainees meet criteria or standards in order to progress within their programs, [5, 7] this may be challenging for programs with multiple training sites and very large trainee cohorts. Trainees may be distributed across numerous settings and rotations, with a variety of teachers and raters of varying teaching ability [25]. Trainees may not always have the same opportunities to practice key skills or receive formative feedback across rotations, settings, and faculty [19, 22]. For instance, a gastroenterology fellow may perform more colonoscopies with a faculty member who offers the trainee more autonomy and feedback, thereby enhancing rates of skill improvement. Random occurrences which lead to larger volumes of high-acuity patients during specific times of year (e. g., increase in trauma during warmer months) can lead to vastly different educational experiences, which in turn can affect assessment performance [19]. Differing training opportunities are often unavoidable, but must be taken into consideration when rotations are reviewed during annual program evaluations and when high-stakes decisions are being made about learners based upon assessment data [14]. Further, trainee opportunities to be observed in clinical settings, considered critical to professional formation and achievement of outcomes, [26] should certainly be examined at a program level to ensure fairness from an equity or comparability standpoint.

When program personnel or committees identify concerns regarding systematic differences in curricula and training experiences for particular trainees, these issues should be brought to the attention of the appropriate program leadership [24]. In the US, members of Clinical Competency Committees are responsible for reviewing trainee progress in relation to Accreditation Council for Graduate Medical Education competencies and specialty specific milestones [16, 27]. Canadian programs will also begin using competence committees to review and make decisions regarding trainees’ progress in meeting key educational milestones [28]. Committees such as these may, at times, recommend targeted experiences for certain trainees to address gaps in training, [16, 28] thereby ensuring fairness with regards to access to educational opportunities.

### Fairness in assessment practices

Just as learning experiences may differ across trainees, assessment opportunities may also differ in unintended ways. One key question programs will need to confront as they synthesize trainee assessment data within competency-based education systems is whether everyone in the program had comparable opportunities (equitable treatment) to be assessed on key skills and behaviours. Are the number of direct observations and assessment opportunities similar for Evette, Viktor, Jose and Suneeta? And are faculty in postgraduate medical education systems actually using criterion-referenced frameworks as they assess trainees [3]? Criterion- or standards-referenced frameworks promote equal treatment across trainees, as all trainees are held to the same standards. Yet, some faculty within competency-based educational systems compare trainees to the ‘best group’ of residents or fellows (norm-referenced) or their own internal benchmarks, which may lead to biased interpretations of observed performance [26, 29–31]. More importantly, is everyone actually being assessed on the same construct or skill? [19, 32] If faculty – our raters – understand a construct differently from its intended meaning, we have introduced measurement error into our assessment processes via construct irrelevant variance, [31–33] which ultimately renders score or assessment interpretations invalid. We also cannot affirm that our trainees are actually receiving equal treatment when it comes to assessment practices [19, 33]. For a construct such as ‘performance in shared decision-making,’ it is possible that our trainees – and even our faculty – may not share a common understanding of the construct being assessed, based upon their own educational, social and cultural backgrounds. Not only do many patient care practices, including patient communication, differ by country of origin, [34] but they may differ by region and even hospital system, where trainees encounter different clinical cultures.

The need for rater training on the use of a particular rating form, the constructs being assessed, and what constitutes competence in specific domains has been recommended to ensure assessment results which accurately capture a trainee’s performance [3, 19, 31, 33, 35, 36]. The widespread use of rating scales is particularly problematic, given the propensity of faculty (raters or assessors in the field) to bring a variety of biases to the task of learner assessment [31, 33, 37]. As Gingerich et al. have pointed out, rater biases may unfortunately even persist despite rater training [37]. Perceptions of trainees during observations can be influenced by a variety of rater biases, [33, 37] including the commonly seen halo error, where a general impression (e. g., ‘great guy!’) influences a rater’s perceptions of a trainee’s performance across all domains [31, 35, 38]. Broad sampling across multiple domains, assessors, and assessment instruments is recommended and can mitigate the impact of extreme ratings (e. g., severity/leniency errors) on a performance assessment [3, 29, 39, 40]. This also allows trainees to demonstrate competence via alternate assessment modalities [22]. Yet, for some training programs – especially very large programs – low return rates for trainee assessments is not uncommon [41]. In such cases, each assessment may carry an inordinate amount of weight during decision-making over trainee competence. A quiet resident who does not speak up during rounds or conferences could potentially – and erroneously – be flagged as deficient, if a faculty rater has incorrectly inferred she has deficits in medical knowledge [21, 29]. Scores derived from instruments, which are dependent upon rater cognition, such as checklists and other rating scales, should be interpreted with great care, especially in ‘low-yield’ assessment environments [14, 30, 35, 42]. In these situations, evidence from other sources (e. g., narrative assessments, portfolio evidence, feedback from faculty, trainees and healthcare professionals who have worked directly with her) can provide additional evidence of a trainee’s progress in meeting progressive goals toward competence and expertise [2, 3, 35, 42, 43].

In the US, Clinical Competency Committee meetings provide a forum where the synthesis and interpretation of postgraduate medical education trainee assessment evidence occurs with the help of committee member input and professional judgment (Table 1). Similar competence committees are being implemented by other countries [3, 28]. The separation of formative assessment, typically by assessors in the field, from decisions concerning progress or promotion is a recommended practice [35]. Members of Clinical Competency Committees or any other assessment oversight committees should be made aware of the limitations of faculty ratings, including the possibility of rater error, when examining and synthesizing learner assessment data [14, 19, 33, 35, 37]. This is especially important for those trainees who are being assessed in a country’s postgraduate training programs for the first time. When committee members have concerns related to inequities in trainee assessment at the program level [22, 24] and/or suspect faculty bias toward trainees (see Fairness in the Learning Environment), clear and timely communication between the committee, the program director and any related residency oversight committee (e. g., Program Evaluation Committee) is essential. A systems or holistic view will ensure that all relevant stakeholders are apprised of these issues [43] and appropriate action can be taken.

**Table 1:** Table of definitions

Term	Definition
Equity	Equity refers to comparability, whether in educational or assessment practices. Fairness from an equity standpoint ensures that trainees have comparable opportunities to *learn*, and then demonstrate new knowledge and skills, given their unique backgrounds [20, 22, 23]. Similar outcomes for subgroups, such as female trainees in male-dominated specialties, would be one indicator of fair treatment from an ‘equity’ perspective [18–20]
Equality	Equality refers to equal practice or treatment [19]. An outgrowth of equality concerns in testing is the use of standardized tests, such as in-training exams and board exams, [19–21] to ensure that all learners in an identical fashion. Fairness from an equality standpoint also ensures that all trainees are treated in a non-discriminatory fashion within learning environments
Competence Committees	Training programs accredited by the Accreditation Council for Graduate Medical Education must establish Clinical Competency Committees, which are responsible for reviewing resident evaluations, assessments, and artifacts, synthesizing all information, and advising program leadership on resident progression related to national competency criteria (milestones) set for all trainees within a specific program [16]. Canadian postgraduate medical education training programs will soon be utilizing competence committees for similar purposes [28]
Milestones	Milestones are competency-based, developmental markers used to determine learner progression through a training program
Program Evaluation Committee	Accreditation Council for Graduate Medical Education training programs are required to establish Program Evaluation Committees, which monitor, maintain, and revise all aspects of the residency program curriculum

### Fairness in decision-making and recommendations

Within competency-based education frameworks, we have moved away from a one-method approach to assessment and now collect information from multiple sources, across multiple contexts [44]. Thus, performance decisions require aggregating assessment information of different types from different sources, [42] both qualitative and quantitative [3, 5]. Recommendations regarding promotion to the next training level may be relatively straightforward when all collected information is consistent. Conflicting information, however, makes deliberations within committees such as Clinical Competency Committees more difficult and requires professional judgment in determining the best way forward [43]. Members of these committees, under the guidance of the chair, will need to determine not only progression toward competency for all trainees in their programs, but when to gather more information, when to delay a recommendation, and when to recommend remediation, probation and dismissal. Regardless, internal decision-making and recommendations about trainees and their progression on competency-based standards needs to be fair, legally defensible [32] and will ultimately depend upon committee members’ expert professional judgment [16, 23]. Programs have also been encouraged to implement systematic approaches when designing and running committees such as Clinical Competency Committees to ensure that reviews of trainee performance relative to milestones or standards yield both valid and legally defensible results [16, 27, 36].

During such reviews, committee members must acknowledge and struggle with the variability in training experiences and assessment data which may influence the fairness of the committee’s decisions and recommendations. Open discussions, where evidence is weighed and all members are able to voice concerns, are key to achieving consensus and delivering defensible recommendations [16, 27]. Fortunately, professional judgment can be supported by standardization of committee processes [27, 43]. While the use of professional judgment in assessment and evaluation decisions has been upheld by US courts, Jamieson et al. [13] stressed the importance of applying consistent expectations to all learners. Arbitrary recommendations and lack of standardization in review processes can make programs vulnerable to legal repercussions [14]. In the US, programs need to be especially careful in adhering to due process, or the ‘guarantee of procedural fairness, [13] when trainees are not meeting expectations and the program is considering remediation, probation, or dismissal proceedings.

### Fairness and legal considerations

Actions such as termination of a postgraduate medical education trainee contract – and even remediation of a trainee – can have a profound impact on the career of a physician in training and may preclude licensure and the ability to become board certified. Thus, dismissal from a training program is often considered to be a career-ending action. Nonetheless, patient safety is paramount and corrective actions may sometimes need to be implemented immediately [36]. While judicial deference is typically given to educational programs to exercise professional judgment regarding trainees’ competency or fitness, [13, 16, 45, 46] programs must still ensure that trainees understand what is expected of them [14] and should treat all trainees in a fair manner [13, 32, 36]. This extends to policies and procedures within a program. Alignment of program policies, procedures and processes with institutional, accreditation system, and national laws or regulations governing fairness in education is advised [36]. Both Canadian and US accreditation agencies [16, 47] require that programs provide due process during disciplinary actions. Fairness within programmatic policies and procedures protects not only the trainee, but also the program, the institution, and ultimately the patient. We use the US as an example in describing due process considerations for postgraduate medical education trainees:

*Procedural due process* generally requires that a trainee be given notice of any deficiency or failure to meet expectations that may result in discipline or termination, an opportunity to examine evidence upon which the academic decision was based, and the opportunity to be heard on the matter, [13] usually through a procedure culminating in an appeal to the highest decision-making authority (often a panel of medical educators who are not directly involved in the matter at hand) [36]. Notice, provided in writing, ensures all parties are operating on the same basis and documents that the trainee was previously aware of expectations (typically competency related), but then failed to meet them. The disciplinary and academic appeals processes should be documented within the institution’s guidelines and shared with all stakeholders (program and trainees).

*Substantive due process* refers to the underlying basis for the action or ‘why’ actions are being taken [13]. Evaluations should be free from bias and grounded in facts [32]. Academic evaluations and disciplinary processes should be based on reasonable and adequate documentation, and should be fair in content and execution [48]. Expectations of trainees should be fair and reasonably consistent, although it is acceptable to assign additional remediation and scrutiny to ensure patient safety.

### Fairness in the learning environment

The learning environment – or environment for learning – includes physical, psychological, social, emotional and relational (e. g., relationships between learners, learners and faculty, and learners, faculty and administration) characteristics of the academic institutions where trainees learn [49–51]. Learning environments have been linked to the ability of trainees to be academically successful as learners [49–51]. Environments that support learning are typically characterized by feedback cultures, where ‘individuals continuously receive, solicit, and use formal and informal feedback’ to enhance performance [52]. In feedback cultures, members at all levels of organizational and educational hierarchies are encouraged to seek out and use feedback for performance improvement [52]. The hallmark of a feedback culture is a psychologically safe learning and/or work environment, [53]where employees or trainees are comfortable seeking out [50, 53] and providing feedback without fear of retaliation. Strict, hierarchical work or learning environments may hinder the type of bi-directional feedback necessary to create feedback cultures [54].

At the postgraduate medical education level, initiatives targeting the learning environment have focused on quality and safety [55] and burnout amongst trainees. Burnout and depression have been correlated with problems in clinical reasoning and medical errors [56]. Both the Accreditation Council for Graduate Medical Education and Royal College of Physicians and Surgeons require that training programs provide safe and supportive learning environments, free from abuse [57, 58]. Unprofessional treatment of trainees has been linked to quality and safety issues in patient care [55]. When trainees believe it is not safe to provide upward feedback to a faculty member, a program director, or even an upper-level resident, patient safety may be compromised.

While it is widely recognized that hostile learning environments are not conducive to trainee well-being and learning, [57, 59–61] a 2014 meta-analysis of 51 studies found that an estimated 59% of our medical education learners (undergraduate and postgraduate) had experienced hostile learning environments involving discrimination and/or harassment, [62]examples of inequitable treatment. In the US, sexual harassment involving primarily female trainees was found to be the most common form of abuse, [62] but lesbian, gay, bisexual and transgender medical students, residents and practising physician survey respondents reported commonly encountering hostile learning and work environments [59, 63, 64]. The Accreditation Council for Graduate Medical Education mandates that all clinical sites must have a standardized process for reporting mistreatment, and residents must be aware of the protocol for reporting mistreatment [55].

## Next steps

Utilizing a systems or holistic approach will allow programs to identify gaps related to fairness, enabling them to enhance the learning environment, provide trainees with equal opportunities to meet targeted milestones, and develop feedback cultures which promote continuous improvement [52]. We recommend that programs consider adding a review of fairness considerations to any programmatic evaluation which is carried out. There are a number of steps educational programs can take to address concerns about fairness highlighted within this paper, including the following:

### Enhancing educational practices to promote fairness

*Capturing themes: *Continuous program improvement cannot occur if problems are not captured in real time, documented, and then acted upon [65]. We recommend that programs document themes which arise in discussions about residents’ progress and communicate those to both the Program Director and other oversight committees within the postgraduate medical education training program. For critical tasks, committees should revisit issues or concerns on a continuous basis with key stakeholders, while tracking progress and noting any pertinent completion due dates. An overhaul of an entire curriculum will obviously take longer than assigning a trainee to a rotation to make up for the lack of a specific training experience [43].

*Feedback loops:* Programs should identify, examine and enhance feedback loops within their assessment systems to allow for better communication and information exchanges between stakeholders [43, 65]. This will allow the competence or assessment oversight committee (e. g., Clinical Competency Committee) to communicate with the program oversight committee (e. g., Program Evaluation Committee) when issues concerning comparability or equity in training experiences come up [43]. Depending upon how often these committees meet, a standardized approach (e. g., emails triggered by committee questions or concerns) to updates may need to be instituted.

### Enhancing assessment practices

Providing faculty development and rater training to all faculty involved in the assessment of your program’s trainees can help to ensure that trainees are being assessed on the constructs of interest [5, 31, 33] and according to standards or criteria. It is not enough to design tools reflecting the specific competency criteria learners should meet, [31, 33]as even relatively simple words can be comprehended differently across individuals [66–69]. To truly establish shared mental models within our assessment system, all stakeholders (faculty, trainees, departmental leadership) who are involved in the assessment of trainees should receive instruction on: what it means to educate and assess within competency-based educational frameworks; how to provide actionable formative feedback; how to properly use and interpret any faculty rating forms in use; and what competence looks like in specific domains [3, 19, 31, 33, 35, 36, 70]. In addition, in large training programs, a group of faculty can be trained to provide faculty development to other faculty (i. e., utilizing train-the-trainer models) [71].

### Enhancing competence decisions

A standardized approach to reviews of trainee progression toward targeted outcomes may help to prevent reviewer biases from affecting the review process and will provide a solid foundation from which professional judgments can be made [27]. We recommend that all committees tasked with reviewing trainee progression (e. g., milestone reviews) develop a standardized approach, such as focusing discussions on evidence related to trainees’ milestone stages, discouraging inferences about trainees, and limiting anecdotal examples not supported by documentation or other assessment evidence [27, 43]. In addition, review templates can help committee members focus on the same criteria across trainees [53].

### Ensuring fairness from a legal standpoint:

All academic decisions should be made based on documented acts, whether omissions, errors, knowledge deficiencies, or inappropriate or unprofessional behaviour in order to ensure fairness. Residents should be informed of deficiencies in language which focuses on performance improvement [49, 72, 73] and is as transparent as possible. Ginsburg et al. found that politeness, including ‘hedging,’ was common in written comments from faculty to residents [70]. As ‘politeness strategies can obscure the intended message’ within feedback, [70] it is critical that written feedback be specific, focus on areas for performance improvement, and be closely aligned with program criteria. We recommend that program directors document all resident deficiencies and, if appropriate, work with the trainee to develop a plan for appropriate remedial action (e. g., mentoring, research, proctoring, root cause analysis) prior to any disciplinary action. Bierer et al. have described an assessment system where the learners themselves take ownership for the remediation process [74].

### Enhancing the learning environment

*Promoting the development of feedback cultures: *If feedback cultures don’t exist within training programs, leadership can start by meeting with faculty and trainees to develop a shared vision for a feedback culture [1, 52]. Faculty *and* trainees will need to be taught what specific, useful feedback looks like, why it is important, how to ask for it, and how to provide it (both verbal and written) [1, 52, 72, 73]. If trainees are resistant to or do not value the feedback provided, they will not act upon it, and further improvement in performance is unlikely [1, 10]. With individual trainees, faculty are advised to offer specific, timely feedback which focuses on reinforcing or modifying behaviours, [73] rather than generic or polite feedback, [1, 70] as the best avenue for improving performance [72]. Establishing a psychologically safe learning environment is paramount, as individuals are less likely to seek feedback if they feel threatened [53]. Advising or coaching learners on how to interpret and use feedback is integral to success [50, 52]. Like any culture change process, this will take time and commitment [43]. At the program level, leadership should not only document feedback collected from trainees, but also report back to trainees when programmatic changes suggested by trainees have been implemented. Departmental and institutional commitment to the development of a feedback culture is critical [52].

*Implement diversity and inclusion training: *Training everyone who comes in contact with learners on what constitutes harassment and discrimination is critical when addressing diversity needs in postgraduate medical education. Learners need to know who to contact if they are subjected to an unprofessional learning environment [55]. We recommend that curricula focusing on diversity and inclusion be adopted and implemented. Many healthcare systems and medical schools now have offices of diversity and/or diversity officers who can act as resources when creating learning experiences which focus on this topic. As sexual discrimination is still commonplace in health professions training, [62] programs targeting racial/cultural diversity may need to be expanded to include sexual harassment and discrimination.

*Dealing with bias and discrimination: *All instances of potential discrimination should be taken seriously, investigated, and remedied as soon as possible [62] Program leadership must take immediate action to investigate allegations [60] when trainees report instances of harassment or discrimination (based on gender, culture/race, sexual orientation, etc.), including belittling remarks, inappropriate comments/jokes, actions, or denial of opportunities [12, 62, 63]. Much work remains to be done in creating safe learning environments [62] and ensuring fair treatment of all trainees, as many, if not most, trainees fail to report harassment or discriminatory practices for fear of reprisals.

## Conclusion

We recognize that the goal of competency-based education systems, in their quest to offer both an outcomes-based framework [5] and a more learner-centred approach, [4] is not to ensure that all trainees have identical clinical opportunities. That said, both equity and equality aspects of fairness play roles in the education and assessment of our trainees. We believe the climate within many postgraduate medical education programs today, where there seems to be some complacency about fairness issues, is reminiscent of the historical treatment of fairness within the fields of testing and measurement [18]. In this paper, we offered an examination of fairness within educational and assessment practices, decision-making, the learning environment, and as a potential legal requirement for training programs. We also offered recommendations to address gaps in fairness (Next Steps). While the existence of assessment oversight or competence committees and annual programmatic evaluations will not guarantee fairness within programs, they can certainly provide a window into ‘hidden’ threats to fairness, [16] as everything from training experiences to assessment practices is examined by these committees. One of the first steps programs can take is to recognize that threats to fairness may exist in any educational program, including their own, [14] and begin conversations with all stakeholders (administration, faculty, trainees, institutional leadership) about how to address these issues.

## Appendix

### Search terms

In a search of the PubMed (PubMed.gov) and Embase databases, the following search terms were utilized:

PubMed:

- (“bias” OR “fairness” OR “equitability” OR “equitable” OR “equity”) AND (“performance assessment” OR “competency based medical education”)
- (“bias” OR “fairness” OR “equitability” OR “equitable” OR “equity”) AND (“medical education”) AND “competency based”
- (“bias” OR “fairness” OR “equitability” OR “equitable” OR “equity”) AND (“medical education”) AND “competency based” AND “post graduate”
- (“bias” OR “fairness” OR “equitability” OR “equitable” OR “equity”) AND (“medical education”) AND “post graduate”
- (“Education, Medical”[Mesh]) AND “Educational Measurement”[Mesh] AND (residency OR “post graduate”) AND (fair OR equity OR equitable) AND competency
- (residency OR “post graduate”) AND (fair OR equity OR equitable) AND “Competency-Based Education”[MeSH Terms]

EMBASE

- “competency based medical education” AND (bias or fairness or equitability or equitable or equity)
- Subject Heading: medical education; keyword: competency AND (bias or fairness or equitability or equitable or equity) AND (“post graduate” OR “post-graduate”)
